# Retinoblastoma incidence and sunlight exposure

**DOI:** 10.1054/bjoc.2000.1215

**Published:** 2000-06

**Authors:** A Jemal, S S Devesa, T R Fears, J F Fraumeni Jr

**Affiliations:** Division of Cancer Epidemiology and Genetics, National Cancer Institute, 6120 Executive Blvd EPS 8049, Rockville, MD, 20892, USA

**Keywords:** retinoblastoma, sunlight, race, economic development, tropical climate, SEER

## Abstract

To evaluate positive findings from an earlier report, we studied the relation between retinoblastoma incidence and ultraviolet (UV-B) radiation levels in the Surveillance, Epidemiology, and End Results (SEER) programme areas of the USA using weighted regression, as well as in international data after adjusting for race, economic development, and climate. The association was not statistically significant within the USA (*P*> 0.20). At an international level, the relation was significant overall and after adjusting for economic development, but it was not significant after adjusting for race and tropical climate, suggesting that environmental factors other than UV-B may be responsible for the geographic patterns of retinoblastoma. © 2000 Cancer Research Campaign


*British Journal of Cancer* (2000) 82(**11**), 1875–1878
© 2000 Cancer Research Campaign
DOI: 10.1054/ bjoc.2000.1215, available online at http://www.idealibrary.com on IDEAL®

### Retinoblastoma incidence and sunlight exposure

**A Jemal, SS Devesa, TR Fears and JF Fraumeni Jr**

Division of Cancer Epidemiology and Genetics, National Cancer Institute, 6120 Executive Blvd EPS 8049, Rockville, MD 20892, USA

**Summary** To evaluate positive findings from an earlier report, we studied the relation between retinoblastoma incidence and ultraviolet (UV-B) radiation levels in the Surveillance, Epidemiology, and End Results (SEER) programme areas of the USA using weighted regression, as well as in international data after adjusting for race, economic development, and climate. The association was not statistically significant within the USA ($P > 0.20$). At an international level, the relation was significant overall and after adjusting for economic development, but it was not significant after adjusting for race and tropical climate, suggesting that environmental factors other than UV-B may be responsible for the geographic patterns of retinoblastoma. © 2000 Cancer Research Campaign

**Keywords**: retinoblastoma, sunlight, race, economic development, tropical climate, SEER

Using retinoblastoma incidence data from different parts of the world, Hooper (1999) reported that rates increased with annual ambient erythemal dose, suggesting that sunlight exposure is a causal factor. He further noted that the relation between retinoblastoma and annual erythemal dose was seen for unilateral but not bilateral tumours. The study included incident cases diagnosed during 1973–1987 in areas participating in the Surveillance, Epidemiology, and End Results (SEER) programme of the US National Cancer Institute. However, the geographic analysis within the USA was based on rates for whites only with total retinoblastoma, while all areas were combined to provide rates among whites and blacks separately by laterality. Many more SEER cases are now available for further evaluation of the incidence patterns for unilateral vs bilateral tumours in the USA.

It is not clear whether the correlation of retinoblastoma incidence with erythemal dose reported at the international level is confounded by race, economic development, and/or climate, especially since retinoblastoma rates in several populations of Africa and Asia are 50–100% higher than in western countries, due apparently to excesses of unilateral tumours that are predominantly non-heritable (Parkin and Stiller, 1995; Stiller and Parkin, 1996). Herein, we examine the relation between retinoblastoma incidence and ultraviolet (UV-B) radiation levels in the SEER areas, and re-analyse the international patterns after accounting for race, economic development, and climate.

### MATERIALS AND METHODS

Based on cases diagnosed during 1973–1995 among children aged 0–14 years, age-adjusted (1970 US population standard) retinoblastoma incidence rates in the USA were computed for each of the nine SEER areas with a population-based cancer registry (Ries et al, 1997; SEER, 1998). Retinoblastoma cases were considered overall for whites and blacks and by laterality for whites. For other US ethnic groups, only the ratios of bilateral to unilateral cases were calculated because annual inter-censal population estimates for these ethnic groups are not available for the SEER areas (personal communication, Statistical Information Office, Population Division, US Census Bureau). For each SEER area, UV-B radiation levels in Robertson-Berger (R-B) units were estimated from a model including latitude, altitude, and cloud cover (Scotto et al, 1996); one minimal erythemal dose is equivalent to about 440 R-B counts. The log-transformed rates for total retinoblastoma and for unilateral vs bilateral tumours among whites were then regressed against log-transformed UV-B exposures, weighted by the inverse of the estimated variance of the log age-adjusted rates (SAS, 1992).

For the international patterns, we used the cumulative retinoblastoma incidence among children aged 0–14 years during 1969–1987 provided by 66 population-based registries around the world as listed in Hooper (1999). We classified registries according to race, economic development, and climate. Registries in Canada, Europe, Australia, and New Zealand were grouped as white, registries in Africa as black, registries in Asia as Asian and registries in the Caribbean, Central and South America as mixed races. Rates for the USA were available for blacks and whites. Areas were categorized as developed or developing following Parkin et al (1999), while areas were classified as tropical or non-tropical based on climatic status as defined by Rosenberg (1999). Hooper regressed the cumulative incidence rates on annual ambient erythemal dose weighted by population. However, data for the registries were collected during time intervals of various lengths, and weighting by population may not be the best approach. For example, the data collection period for Kuwait was 9 years during 1974–1982, whereas for Brazil (Fortaleza) it was 3 years during 1978–1980. In addition, when data include relatively small and large values, spanning several log scales, it is customary to use transformed values during analysis (Dowdy and Wearden, 1991). Therefore, we regressed the log-transformed cumulative incidence rates on log-transformed annual ambient erythemal dose weighted by the number of cases, which approximates the inverse of the estimated variance. We then fitted the log-transformed incidence rates on race, economic development and climate to assess their impact on the international variation in retinoblastoma incidences independent of erythemal dose. We then re-examined the relationship of erythemal dose and retinoblastoma incidence after adjusting for the confounding factors.

*Received 28 September 1999*
*Revised 6 January 2000*
*Accepted 6 January 2000*

*Correspondence to:* A Jemal

**Table 1** Incidence of retinoblastoma (per 100 000 person-years) among children (0–14 years) in the nine SEER areas of the USA, 1973–1995

| Area | Annual R-B[a] count × $10^{-4}$ | PYR[c] | White | | | | | | | | Black | |
|---|---|---|---|---|---|---|---|---|---|---|---|---|
| | | | Unilateral | | Bilateral | | Unspecified laterality | | Total retinoblastoma | | Total retinoblastoma | |
| | | | Count | Rate | Count | Rate | Count | Rate | Count | Rate | Count | Rate |
| Seattle | 94 | 12610738 | 42 | 0.30 | 20 | 0.14 | 0 | 0.00 | 62 | 0.44 | 3 | 0.36 |
| Detroit | 99 | 15095502 | 39 | 0.25 | 13 | 0.08 | 0 | 0.00 | 52 | 0.33 | 30 | 0.46 |
| Connecticut | 108 | 13315287 | 20 | 0.14 | 11 | 0.08 | 6 | 0.04 | 37 | 0.26 | 4 | 0.22 |
| Iowa | 119 | 14398475 | 51 | 0.33 | 14 | 0.09 | 0 | 0.00 | 65 | 0.42 | 1 | 0.27 |
| Utah | 133 | 10832810 | 33 | 0.25 | 11 | 0.08 | 1 | 0.01 | 45 | 0.35 | 0 | 0.00 |
| Atlanta | 145 | 5713017 | 15 | 0.24 | 5 | 0.08 | 0 | 0.00 | 20 | 0.32 | 16 | 0.42 |
| San Francisco[b] | 147 | 10617117 | 30 | 0.25 | 14 | 0.11 | 0 | 0.00 | 44 | 0.36 | 10 | 0.37 |
| New Mexico | 193 | 7100491 | 20 | 0.25 | 5 | 0.06 | 1 | 0.01 | 26 | 0.33 | 0 | 0.00 |
| Hawaii | 246 | 1264889 | 6 | 0.40 | 1 | 0.07 | 0 | 0.00 | 7 | 0.46 | 2 | 1.02 |
| Total | | 90948326 | 256 | 0.26 | 94 | 0.09 | 8 | 0.01 | 358 | 0.36 | 66 | 0.40 |

Rates are age-adjusted using the 1970 US population as the standard. [a]A count of about 440 R-B units = 1 minimal erythema dose (MED). [b]Including Oakland. [c]PYR = person-years.

**Table 2** Coefficients for relationships of UV-B and retinoblastoma among whites, SEER areas 1973–1995

| Laterality | Slope | *P*-values | $R^{2a}$ |
|---|---|---|---|
| Unilateral | 0.11 | 0.7804 | 0.0118 |
| Bilateral | –0.53 | 0.2296 | 0.1983 |
| Total retinoblastoma | –0.07 | 0.8122 | 0.0086 |

[a]The proportion of variation in retinoblastoma incidence explained by annual erythemal dose and other explanatory variables in the model.

### RESULTS

In our survey of retinoblastoma in the USA, a total of 358 cases of retinoblastoma were diagnosed among white children and 66 cases among black children during 1973–1995 in the nine SEER areas (Table 1). The total retinoblastoma incidence rate was similar among whites and blacks, 0.36 and 0.40 cases per 100 000 person-years, respectively. The rates for total retinoblastoma ranged from 0.26 per 100 000 person-years in Connecticut to 0.46 in Hawaii among whites, and from 0.00 in New Mexico and Utah to 1.02 in Hawaii among blacks. The rates in Hawaii were based on only seven cases in whites and two in blacks. Overall, the incidence of bilateral retinoblastoma was higher among blacks (0.14 cases per 100 000, based on 23 cases) than whites (0.09), though not statistically significant ($P > 0.1$). In contrast, the incidence of unilateral tumours was the same (0.26) among blacks and whites. The bilateral/unilateral case ratios were 0.37 for whites, 0.53 for blacks, 0.27 for Asians, 0.11 for American Indians, and 0.33 for other or unreported ethnicity (based on 350, 66, 33, 10, and 8 cases, respectively). Table 2 summarizes the regression of the log-transformed retinoblastoma incidence by laterality on log-transformed UV-B among whites in SEER areas. No significant relationship with UV-B exposure ($P > 0.20$) was seen for any of the retinoblastoma categories, and UV-B explained less than 1% of the variation in total retinoblastoma incidence.

Based on the international data, we replicated Hooper's regression of the rates on erythemal dose (Table 3). The regression of log-transformed retinoblastoma incidence on log-transformed annual erythemal dose was positive and statistically significant ($P = 0.0014$, $R^2 = 0.1492$). In univariate analyses, race was significant ($P = 0.0129$, $R^2 = 0.1585$), as was climate ($P = 0.0008$, $R^2 = 0.1614$) and economic development ($P = 0.0147$, $R^2 = 0.0895$) (data not shown). The erythemal dose coefficient was significant ($P = 0.0379$) after adjusting for economic development, but was not significant ($P > 0.05$) after adjusting for race, climate or the three factors simultaneously. The erythemal dose coefficient decreased from 0.23 to 0.14 as the $R^2$ increased from 0.1492 to 0.2547 with adjustment for the three factors. Notable were the elevated rates in areas characterized by a predominance of blacks

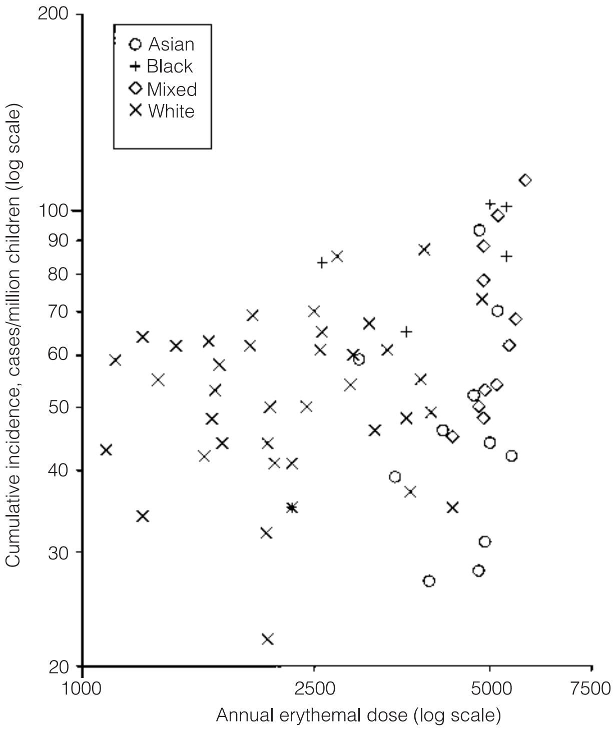

**Figure 1** Distribution of retinoblastoma incidence among children (0–14 years) according to race and annual erythemal dose

© 2000 Cancer Research Campaign

**Table 3** Regression results for the relationship of retinoblastoma and annual erythemal dose at the international level, based on data from 66 registries

| Model | Weight | Slope | *P*-values | $R^{2a}$ |
|---|---|---|---|---|
| Linear–linear | Population | 0.004 | 0.0076 | 0.1062 |
| Log–log | | | | |
| Unadjusted | Count | 0.23 | 0.0014 | 0.1492 |
| Adjusted for: | | | | |
| Economic development | Count | 0.26 | 0.0379 | 0.1501 |
| Race | Count | 0.17 | 0.1445 | 0.1876 |
| Tropical climate | Count | 0.11 | 0.3657 | 0.1723 |
| All three | Count | 0.14 | 0.2755 | 0.2547 |

[a]The proportion of variation in retinoblastoma incidence explained by annual erythemal dose and other explanatory variables in the model. Based on data reported in Hooper (1999)

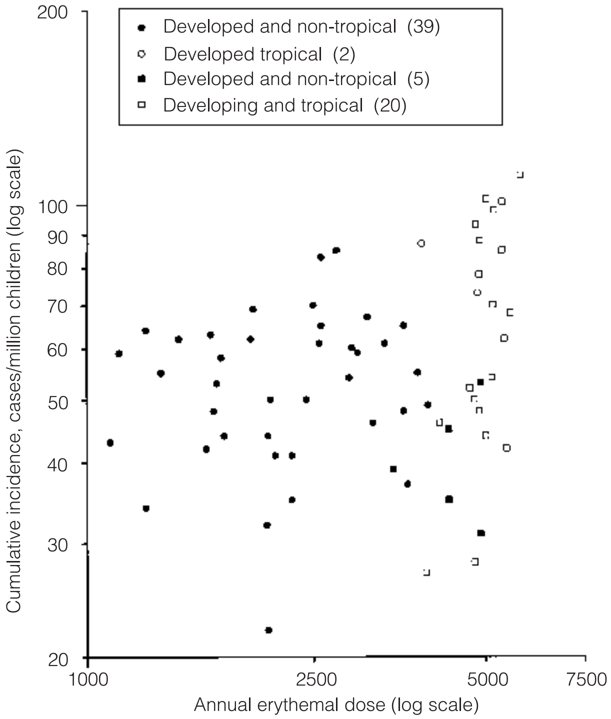

**Figure 2** Distribution of retinoblastoma incidence among children (0–14 years) according to tropical climate, economic development, and annual erythemal dose or mixed races (Figure 1), and the spread of rates according to economic development and climate (Figure 2). Race and climate explained a greater proportion of the variation in incidence than erythemal dose.

### DISCUSSION

The absence of a UV-B gradient for retinoblastoma incidence in the SEER areas of the USA stands in contrast to the patterns described for sunlight-related cancers of the skin (Fears et al, 1976; Scotto et al, 1996), uveal tract (Tucker et al, 1985) and conjunctiva (Sun et al, 1997). Furthermore, our analysis of the international patterns of retinoblastoma suggests that tropical climate and exposure related to ethnic susceptibility and economic development may have confounded the association that Hooper (1999) reported with sunlight exposure. Our estimates of the slope and *P*-value using Hooper's method agree with his report of a significant erythemal dose gradient for retinoblastoma. When we used observed counts as a weight in a log–log model, the relationship remained significant, but it became nonsignificant after adjusting for economic development, race and tropical climate. In view of the elevated rates for unilateral retinoblastoma reported in a number of developing countries, particularly in tropical regions, attention should be given to the possible role of infectious agents or environmental exposures other than sunlight that are prevalent in these areas (Stiller and Parkin, 1996).

There are several models that can be used to estimate UV radiation, and variations in estimated values by these different methods are known to exist (Diffey and Elwood, 1994). However, the estimates for the SEER areas by the method we employed and by the method used by Hooper (1999) were highly correlated (Pearson correlation coefficient = 0.95, $P = 0.0003$). Moreover, the relationship between retinoblastoma incidence and UV-B radiation levels in the SEER areas was not affected by the differences in methods.

In contrast to reports that unilateral retinoblastoma is more common in black than white children in the USA (Kramer et al, 1983; Stiller and Parkin, 1996), an earlier report using 1974–1985 SEER data (Tamboli et al, 1990) and our updated analysis of SEER data through 1995 reveals that the incidence of unilateral tumours is similar in blacks and whites, while the incidence of bilateral tumours is non-significantly higher in blacks. Although the patterns of unilateral retinoblastoma in the USA have shown little variation by region or ethnic group, there is some indication that socioeconomic factors may play a role (Bunin et al, 1989) as in other parts of the world.

### ACKNOWLEDGEMENTS

We are grateful to all staff participating in the SEER registries.

### REFERENCES

Bunin GR, Meadows AT, Emanuel BS, Buckley JD, Woods WG and Hammond GD (1989) Pre- and postconception factors associated with sporadic heritable and nonheritable retinoblastoma. *Cancer Res* **49**: 5730–5735

Diffey BL and Elwood JM (1994) Tables of ambient solar ultraviolet radiation for use in epidemiological studies of malignant melanoma and other diseases. In: *Melanoma Epidemiology*, Gallagher RP and Elwood JM (eds), pp. 81–105. Kluwer: Boston

Dowdy S, Wearden S (1991) *Statistics for research*. John Wiley & Sons: New York

© 2000 Cancer Research Campaign

Fears TR, Scotto J and Schneiderman M (1976) Skin cancer, melanoma, and sunlight. *Am J Public Health* **66**: 461–464

Hooper ML (1999) Is sunlight an aetiological agent in the genesis of retinoblastoma? *Br J Cancer* **79**: 1273–1276

Kramer S, Meadows AT, Jarrett P and Evans AE (1983) Incidence of childhood cancer: experience of a decade in a population-based registry. *J Natl Cancer Inst* **70**: 49–55

Parkin DM and Stiller CA (1995) Childhood cancer in developing countries: environmental factors. *Int J Pediat Hematol Oncol* **2**: 411–417

Parkin DM, Pisani P and Ferlay J (1999) Global cancer statistics. *CA Cancer J Clin* **49**: 33–64

Ries LAG, Kosary CL, Hankey BF, Miller BA, Harras A and Edwards BK (eds) (1997) *SEER Cancer Statistics Review, 1973–1994*, NIH Publ. No. 97-2789. National Cancer Institute: Bethesda, MD

Rosenberg MT (1999) Geography@About.com (Online)

SAS Institute Inc (1992) SAS/STAT User's Guide, Version 6, Vol. 2, 4th Edn. SAS Institute Inc: Cary, NC

Scotto J, Fears TR and Fraumeni JF Jr (1996) Solar radiation. In: *Cancer Epidemiology and Prevention*, Schottenfeld D and Fraumeni JF Jr (eds), pp. 355–372. Oxford University Press: New York

SEER (Surveillance, Epidemiology, and End Results) Program, Public Use CD-ROM 1973–1995 (1998) National Cancer Institute, Surveillance Program

Stiller CA and Parkin DM (1996) Geographic and ethnic variations in the incidence of childhood cancer. *Br Med Bull* **52**: 682–703

Sun EC, Fears TR and Goedert JJ (1997) Epidemiology of squamous cell conjunctival cancer. *Cancer Epidemiol Biomarkers Prev* **6**: 73–77

Tamboli A, Podgor MJ and Horm JW (1990) The incidence of retinoblastoma in the United States: 1974 through 1985. *Arch Ophthalmol* **108**: 128–132

Tucker MA, Shields JA, Hartge P, Augsburger J, Hoover RN and Fraumeni JF Jr (1985) Sunlight exposure as risk factor for intraocular malignant melanoma. *N Engl J Med* **313**: 789–792

*© 2000 Cancer Research Campaign*
